# Bottom Sediments as Dynamic Arenas for Anthropogenic Pollutants: Profiling Sources, Unraveling Fate Mechanisms, and Assessing Ecological Consequences

**DOI:** 10.3390/ijms262010219

**Published:** 2025-10-21

**Authors:** Abdullah Maqsood, Ewa Łobos-Moysa

**Affiliations:** Department of Water and Wastewater Engineering, Faculty of Energy and Environmental Engineering, Silesian University of Technology, Akademicka 2A Str., 44-100 Gliwice, Poland; abdullahmaqsood414@gmail.com

**Keywords:** bottom sediments, anthropogenic pollutants, fate and transformation, redox processes, microbial biodegradation

## Abstract

Bottom sediments play a central role in regulating contaminant dynamics in aquatic systems. They act as both storage sites and reactive zones where contaminants undergo transformation, sequestration, or remobilization. Contaminants primarily enter sediments through anthropogenic activities, including agricultural runoff, industrial effluents, wastewater discharge, urban runoff, and mining operations. This review focuses on six major contaminant groups, including nutrients, heavy metals, pharmaceutical residues, pesticides, polycyclic aromatic hydrocarbons, and microplastics, and examines the mechanistic processes that govern their fate in sediments. The main mechanisms includesorption–desorption on minerals and organic materials, sedimentation, and redox processes that regulate metal immobilization and sulfide formation. The persistence and mobility of contaminants are also influenced by synergistic or antagonistic interactions among pollutants, microbial transformation of organic compounds, and oxidative degradation of microplastics by reactive oxygen species. Contaminants can affect benthic communities by causing toxic effects and oxygen depletion. They also may alter microbial and macrofaunal populations and contribute to bioaccumulation and biomagnification. Ultimately, these insights are important for predicting contaminant behavior and assessing ecological risks, which directly informs the development of effective environmental monitoring programs and sustainable sediment remediation strategies for the long-term protection of aquatic ecosystems.

## 1. Introduction

Aquatic ecosystems around the world are increasingly threatened by the accumulation of man-made contaminants [1,2,3,4,5,6]. When these contaminants enter aquatic environments, they are commonly deposited in bottom sediments, which play a major role in determining their long-term fate [7,8,9]. Bottom Sediments pollution is caused by wastewater discharge, urbanization, agriculture, and industrial activities, which affects ecosystem stability and water quality [10,11,12,13,14]. As a result, water quality may decline, eutrophication can be accelerated, and biodiversity may decrease in both freshwater and marine ecosystems [15,16]. The interactions between sediments, overlying water, and benthic communities can further intensify impacts, altering nutrient cycling, contaminant partitioning, and food-web functioning [17,18,19]. In addition to affecting ecosystems, contaminated sediments may also threaten human health, decrease food safety, and increase water treatment costs [19]. These effects make contaminated sediments an important concern for global environmental management and policy.

Numerous contaminants, such as nutrients, pesticides, heavy metals (HMs), pharmaceutical active compounds (PhACs), polycyclic aromatic hydrocarbons (PAHs), and microplastics (MPs), show different but interconnected behaviors in sediments [20,21,22]. Nutrients may be temporarily sequestered within sediments but can later be remobilized, causing internal eutrophication and reducing oxygen levels [16,23]. The HMs are often persistent in sediments but undergo redox-sensitive transformations that determine whether they stay immobilized or become remobilized [24,25,26,27]. Pesticides and PhACs are generally resistant to biodegradation and can form transformation products that may remain persistent in sediments [11,28,29]. PAHs strongly bind to organic-rich sediment particles, while MPs degrade very slowly, resist chemical breakdown, and can transport co-contaminants [30,31,32,33]. Together, these groups create complex contaminant mixtures, with potential combined impacts on benthic organisms, sediment-associated flora, and aquatic food webs that are difficult to fully predict.

Even though we know a lot about where contaminants occur and how they are distributed, we still do not fully understand how they transform, persist, or affect ecosystems. Key gaps include how multiple pollutants interact, how redox processes and microbes transform persistent compounds, and how contaminants cycle over the long term in natural sediments. These uncertainties matter because contaminants may harm benthic animals, reduce the growth and photosynthesis of sediment-associated plants, and accumulate up the food chain, posing potential risks to the entire aquatic ecosystem. This review therefore aims to (i) provide an integrated overview of the major sources and pathways by which contaminants reach bottom sediments, (ii) synthesize the physical, chemical, and microbial processes that regulate their transformation, retention, and release, (iii) evaluate how interactions among co-occurring contaminants shape their persistence, mobility, and ecological impacts, and (iv) highlight critical research gaps and management challenges to advance sediment-focused risk assessment and remediation strategies. To provide a structured and comparative overview, the mechanistic pathways, concentration ranges, evidence strength, and risk relevance for these six contaminant groups are synthesized into a risk-translation element.

## 2. Review Methodology

We conducted a thorough literature search using Scopus, Web of Science, Google Scholar, and PubMed for the period 2008–2025, with keywords such as “Bottom Sediments,” “Anthropogenic Pollutants,” “Aquatic Environments,” “Aquatic Pollution,” “Fate and Transformation,” “Sorption Adsorption,” “Redox Processes,” “Microbial Biodegradation,” and “Benthic Community.” The initial search identified 709 articles, which were carefully screened for relevance. Titles, abstracts, and full texts were reviewed, and studies were included if they were in English, peer-reviewed, focused on sediments, and provided information on anthropogenic pollutants, their impacts on benthic communities, or the mechanisms of their occurrence, migration, and transformation. After this process, 167 articles were selected, comprising 113 original research papers and 54 review articles.

**Figure 1:**
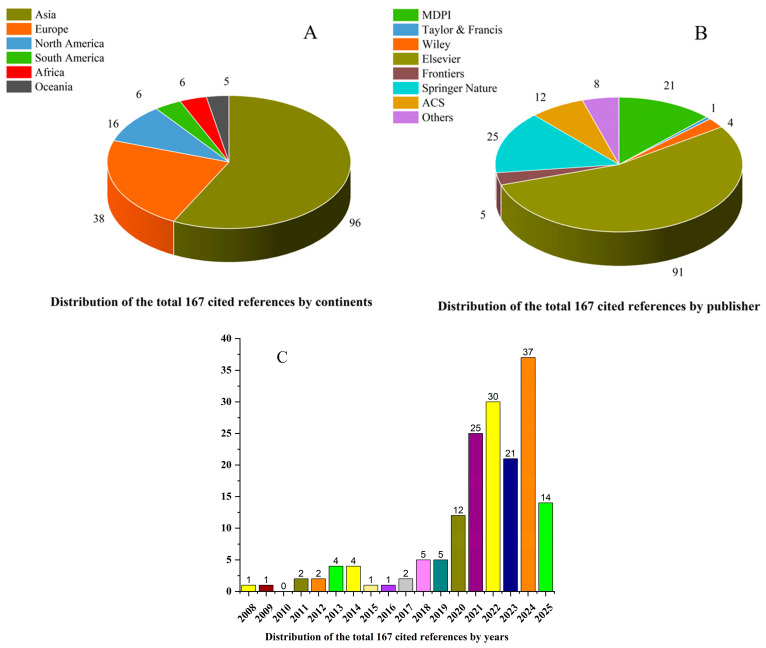
Bibliometric overview of the 167 cited references used in this review: (**A**) distribution by continent, (**B**) distribution by publisher, and (**C**) distribution by publication year (2008–2025).

The selected references were analyzed for their distribution by continent, publisher, and year, providing an overview of the sources included in this review. Most studies originated from Asia, Europe, and North America (Figure 1A). In terms of publication venue, the majority were published in Elsevier journals, followed by Multidisciplinary Digital Publishing Institute (MDPI), Springer Nature, American Chemical Society (ACS), Frontiers, Wiley, Taylor & Francis, and other less common publishers (Figure 1B). The cited studies span the period 2008 to 2025, showing the years in which the included studies were published (Figure 1C).

## 3. Pollutant Profile and Pathways of Input in Bottom Sediments

**Figure 2:**
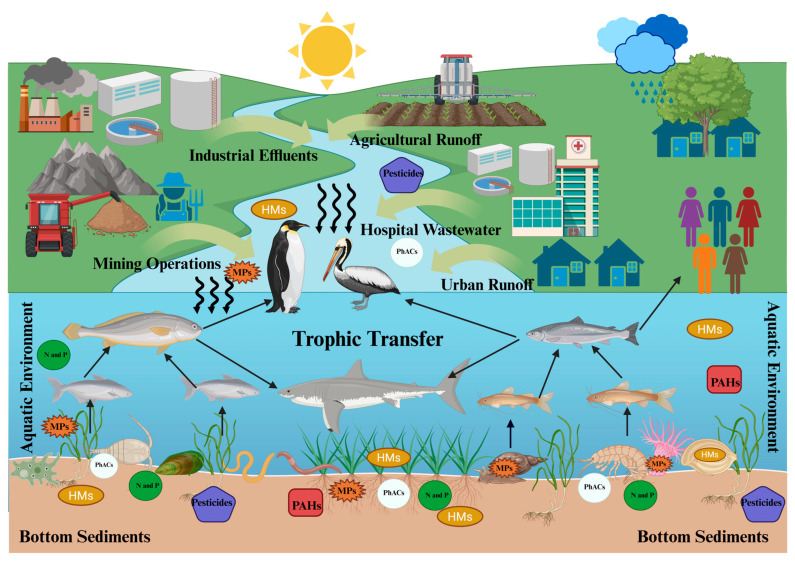
Conceptual framework illustrating the key anthropogenic pathways of pollutants into bottom sediments and the resulting ecological impacts. Created in BioRender. Maqsood, A. (2025) https://BioRender.com/ydq3erq (accessed on 15 October 2025).

Anthropogenic pollutants that accumulate in sediments can be broadly classified into several categories, including nutrients, HMs, organic compounds, and MPs. These pollutants enter the sediments through both point and non-point sources (as illustrated in Figure 2). Understanding the types of pollutants present in sediments is critical for assessing their potential risks to aquatic ecosystems and human health. In this section, we provide a detailed overview of the main pollutant groups commonly found in bottom sediments, highlighting their typical concentrations and sources.

### 3.1. Nutrients: Nitrogen and Phosphorus

Nitrogen (N) and phosphorus (P) are essential nutrients that regulate primary productivity in aquatic systems by supporting the growth of plants, microbes, and algae, as well as overall biodiversity [34,35,36]. In eutrophic environments, these nutrients exist in bottom sediments as both inorganic and organic forms, with organic forms potentially making up a significant proportion [37].

Phosphorus is a key nutrient for aquatic productivity, but excessive inputs may lead to eutrophication, resulting in algal blooms and hypoxia [15,38]. Its concentration in sediments is influenced by factors such as sediment composition, water depth, and oxygen conditions at the sediment–water interface [12,39]. Phosphorus enters sediments from external (allochthonous) sources, including natural runoff, agricultural activities, industrial discharges, and domestic wastewater, as well as internal (autochthonous) sources from sediment-bound nutrient stores [3,40,41], as summarized in Table 1.

Nitrogen in bottom sediments originates from multiple sources. Point sources include effluents from municipal wastewater treatment plants (WWTPs), industrial discharges, intensive livestock farming (e.g., cattle, poultry, swine), and aquaculture operations [3,36,41]. Non-point sources consist of diffuse inputs such as agricultural runoff, urban stormwater, wetland drainage, and leaching from septic systems [2,41,42], as also summarized in Table 1.

### 3.2. Mineral Compounds

The HMs are frequently detected in bottom sediments, where they act as critical environmental pollutants [13,43]. Elements such as lead (Pb), mercury (Hg), cadmium (Cd), arsenic (As), chromium (Cr), copper (Cu), and zinc (Zn) are commonly reported in bottom sediments across various regions [3,44]. While some metals such as Fe, Cu, Zn, and Mn are biologically essential, others, particularly Hg, Pb, and Cd, are toxic even at trace levels [45,46]. Research indicates that nearly 90% of HMs in sediments are associated with fine particles, typically in the range of 0.2 to 20 μm [47,48]. These metals remain in the environment because they cannot degrade and mainly originate from human activities such as industrial, urban development, and agricultural sources [13,45,49].

Several industrial activities play a significant role in introducing HMs into sediments. These include mining, smelting, electroplating, cement and steel manufacturing [26,50,51], leather tanning, dye production, and fossil fuel combustion [13,46]. Other contributors include agrochemicals, including phosphate-based fertilizers, pesticides, fungicides, algicides [26,50,52], and antifouling agents, as well as urban runoff, and wastewater discharges, which can further increase HMs contamination [46,51,52]. For example, approximately 70% of environmental As originates from human activities, with global sediment concentrations typically averaging around 5 mg/kg [44].

Several regional studies highlight the extent and sources of HM contamination in bottom sediments. In the Payra River, Bangladesh, sediment concentrations were reported as As (4.70 ± 0.72 mg/kg), Cd (0.10 ± 0.03 mg/kg), Hg (0.28 ± 0.21 mg/kg), Pb (14.60 ± 5.56 mg/kg), Cu (34.07 ± 6.84 mg/kg), Cr (44.59 ± 8.98 mg/kg), and Zn (66.42 ± 12.86 mg/kg), mainly originating from coal combustion, industrial discharges, mining, leather tanning, and agricultural runoff [13]. In Canada, Hg concentrations of 0.032–0.063 mg/kg in sediments from the Beaufort Sea were largely linked to oil exploration, chlor-alkali plant operations, drilling, and atmospheric deposition [53].

Consistent patterns of HM accumulation have also been reported in European and Asian freshwater systems. In the Odra River estuary, Poland, Kucharski et al. [3] reported As (1–46 mg/kg), Cd (0.2–13 mg/kg), Pd (1–217 mg/kg), Cu (1–252 mg/kg), Zn (6–2114 mg/kg), and Hg (0.01–2.40 mg/kg), were associated with mining and mineral processing, industrial effluents, municipal waste discharge, anti-corrosion coatings and antifouling paints. Similarly, In the Upper Silesian Industrial Region, Poland, Rzetala et al. [44] observed Cu concentrations ranging from 11 to 298 mg/kg and Zn from 142 to 35,300 mg/kg, primarily due to Cu mining and coal mine drainage effluents. In Dal Lake, Jammu Kashmir, Saleem et al. [43] found Pb ranging from 129.85 to 272.10 mg/kg, Cd (8.81–10.54 mg/kg), and Zn (145.02–170.52 mg/kg), resulting from automobile emissions, improper disposal of Pb-containing products, and electronic waste. In Egypt, El-Saadani et al. [45] recorded Hg concentrations of 0.16 mg/kg in Nile River sediments, associated with brick and cement manufacturing. In various freshwater systems across the United States, Paul et al. [51] found Pb concentrations in sediments ranging from 6 to 14 mg/kg and Cr from 6 to 104 mg/kg, primarily from agricultural inputs like fertilizers and pesticides (for Pb), and industrial activities such as tanneries and steel manufacturing (for Cr). Lastly, in Southeast Asia, elevated Pb levels (5.36–6.87 mg/kg) were recorded in sediments from the Dasun Estuary, Indonesia, driven by industrial discharges and increasing tourism pressure [49]. These studies collectively demonstrate that HMs contamination in sediments is widespread, with concentration and composition generally reflecting local industrial, agricultural, and urban activities.

### 3.3. Microplastics

MPs are emerging as major pollutants worldwide, with the aquatic environment serving as a primary sink [1,14]. Since the mid-20th century, global MP production has crossed 400 million tons [54]. Around 10% of municipal solid waste worldwide consists of plastics, yet only a small portion (6 to 14%) is recycled or recovered, with most being discarded without proper disposal [55]. MPs are widely distributed in coastal waters, the water column, and sediments, which act as major reservoirs for their accumulation [1,56]. Studies indicate that 70–90% of MP particles are accumulated in sediment profiles [57].

Plastic fragments are generally classified into five categories based on size: nanoplastics (NPs) (1 to 100 nm), MPs (<5 mm), mesoplastics (5–25 mm), macroplastics (>25 mm), and megaplastics (>100 mm) [8,54]. Based on origin, MPs are divided into two types: primary and secondary. Primary MPs are manufactured at small sizes for use in cosmetics, industrial abrasives, coatings, and pharmaceuticals, often entering aquatic systems via wastewater [1,58]. In contrast, Secondary MPs result from the degradation of larger plastic items (e.g., bottles, bags, fishing gear) through mechanical fragmentation, photodegradation, thermal stress, and microbial activity [1,8]. According to research, about 69–81% of the plastic pollutants found in marine environments may be secondary MPs [8].

Anthropogenic sources of MPs in sediments include urban runoff, wastewater effluents (including treated wastewater), and stormwater discharges. Agricultural practices, such as plastic mulching and polymer-coated fertilizers, also contribute to MP pollution [1,58,59]. Particularly, wastewater discharge alone contributes an estimated 38.5 × 10^15^ MP particles annually in aquatic environments [60]. Other sources include tire wear particles, synthetic textile fibers, landfill leachate, and poorly managed plastic waste [1,8]. Marine-based sources come from shipping, offshore oil and gas activities, illegal waste dumping, and fisheries related debris such as nets and ropes. Coastal tourism and recreational boating also add additional MP loads [56,61]. According to research about 75–90% of MPs may come from land-based activities, while 10–25% may come from marine sources [56,62].

MPs in sediments are composed of many different polymers. The most common are polyethylene (PE), polypropylene (PP), polystyrene (PS), polyvinyl chloride (PVC), polyurethane (PUR), polyethylene terephthalate (PET), and nylon [58,59]. These polymers together account for nearly 90% of global plastic production. Among them, PE (36%) and PP (21%) are the most widely produced and are also the most common in aquatic environments [8]. The transport and deposition of MPs in sediments depend on polymer properties such as size, shape, density, and crystallinity, as well as environmental factors like sediment composition, hydrodynamics, redox potential, and bioturbation. These factors together control processes such as aggregation, biofouling, sedimentation, and resuspension [8,63]. Detailed data on the sources, abundance, and characterization of MPs are presented in Table 2.

### 3.4. Organic Compounds

Organic compounds in bottom sediments form a complex mixture of pollutants with different chemical properties, environmental behaviors, and ecological effects [64]. The main organic pollutants in bottom sediments are agricultural pesticides, PhACs, and PAHs. They are important because of their persistence and toxicity. These compounds mainly come from human activities such as pharmaceutical use, agricultural practices, and industrial processes [4,64].

#### 3.4.1. Pharmaceutical Active Compounds

The PhACs are a different group of biologically active substances used in human and veterinary medicine. They are applied to treat infections, reduce pain and inflammation, manage neurological disorders, and address chronic diseases [65,66,67]. This group includes antibiotics, non-steroidal anti-inflammatory drugs, and anticonvulsants. Their different physicochemical properties influence how persistent they are and how strongly they bind to sediments [4,66,68]. Many PhACs are poorly biodegradable, environmentally stable, and inefficiently removed by conventional WWTPs. As a result, they may be continuously released into aquatic systems [11,66].

PhACs are widely used and not fully removed during treatment, they are now consistently found in bottom sediments around the world. For example, Kucharski et al. [68] reported several PhACs in bottom sediments of the Odra River estuary, Poland: carbamazepine (0.0798 mg/kg), diclofenac (0.0494 mg/kg), clindamycin (0.0877 mg/kg), nalidixic acid (0.0569 mg/kg), lincomycin (0.0198 mg/kg), ofloxacin (0.0518 mg/kg), and norfloxacin (0.0457 mg/kg), mainly originating from WWTPs and industrial effluents. Similarly, Pizzini et al. [4] found diclofenac (0.253 mg/kg), azithromycin (6.000 mg/kg), erythromycin (3.524 mg/kg), and clarithromycin (5.366 mg/kg) in sediments. Hospital waste and WWTP discharge were identified as the main sources. In the Danube River at Budapest, Kondor et al. [69] observed carbamazepine concentrations of up to 0.3959 mg/kg. Similarly, Nantaba et al. [65] reported various PhACs in sediments of Lake Victoria, Uganda. These included carbamazepine (0.024 mg/kg), ibuprofen (0.050 mg/kg), diclofenac (0.0089 mg/kg), levofloxacin (0.130 mg/kg), ciprofloxacin (0.120 mg/kg), enoxacin (0.075 mg/kg), sulfamethoxazole (0.014 mg/kg), oxytetracycline (0.030 mg/kg), and erythromycin (0.038 mg/kg). The contamination was mainly linked to pharmaceutical effluents, poultry farms, and agricultural runoff.

#### 3.4.2. Agricultural Pesticides

More than three million tons of pesticides are used globally each year, with China leading in consumption, followed by the United States, Argentina, Thailand, Brazil, Italy, France, Canada, Japan, and India [10]. Pesticides are chemical agents used to control, eliminate, or inhibit weeds, insects, and fungal diseases that threaten crops and agricultural productivity [70]. Their persistence and widespread use raise concerns about the contamination of aquatic systems, especially sediments [71]. Their sources include agricultural runoff, irrigation return flows, atmospheric deposition, industrial discharges, WWTP effluents, stormwater runoff, groundwater leaching, and improper disposal [5,10,71].

Pesticide contamination in bottom sediments has been widely documented, with multiple studies highlighting the occurrence of herbicides, insecticides, and fungicides in agricultural regions. In the Tandilia mountain system streams, Argentina, San Juan et al. [71] detected atrazine (0.002–0.057 mg/kg), acetochlor (0.0086–0.0326 mg/kg), 2,4-D (0.0391–0.0461 mg/kg), chlorpyrifos (0.0008–0.0505 mg/kg), and cypermethrin (0.0001–0.0205 mg/kg) in sediments, primarily introduced through surface runoff, leaching, and spray drift from nearby soybean, corn, sunflower, wheat, and barley crops. Similarly, Peris et al. [5] reported bentazone (0.00257–0.232 mg/kg), oxadiazon (0.0570–1.252 mg/kg), cypermethrin (0.004–0.327 mg/kg), and chlorpyrifos (0.0008–0.274 mg/kg), in sediments from rice cultivation areas in NE Spain, reflecting continuous inputs from intensive agricultural practices. Mac Loughlin et al. [70] observed high levels in La Plata, Argentina: acetochlor (4.315 mg/kg), glyphosate (1.146 mg/kg), AMPA metabolite (4.032 mg/kg), trifluralin (0.740 mg/kg), atrazine (0.183 mg/kg), chlorpyrifos (2.258 mg/kg), λ-cyhalothrin (0.649 mg/kg), and epoxiconazole (0.652 mg/kg), attributed to surface runoff from horticultural zones. These findings suggest widespread and diverse pesticide inputs that may affect sediment quality in agriculturally influenced environments.

#### 3.4.3. Polycyclic Aromatic Hydrocarbons

The PAHs are persistent organic compounds made of fused benzene rings [72]. These compounds can persist in the environment, accumulate in organisms, and cause serious effects including toxicity, mutagenicity, and carcinogenicity [73]. The main sources of PAHs are petrogenic inputs, such as petroleum leaks and urban runoff, and Pyrogenic inputs come from incomplete burning of fuels like coal, petroleum, and biomass, as well as from fossil fuel based industrial processes [72,74].

Many studies have reported PAHs concentration and profiles in bottom sediments worldwide, identifying different sources and levels of contamination. For example, Baran et al. [75] found PAHs in the Rożnów Dam Reservoir, Poland, with concentrations ranging from 0.00805 to 0.980 mg/kg. The PAHs were dominated by 4-ring compounds, including pyrene, phenanthrene, fluoranthene, benz[a]anthracene, benzo[k]fluoranthene, benzo[b]fluoranthene, and chrysene, mainly originating from fossil fuel combustion and petroleum inputs. According to Skic et al. [74] sediments from the Rybnik Dam Reservoir, Poland, contained 0.4989–24.3217 mg/kg of PAHs. The contamination was dominated by high molecular weight PAHs (>4 rings), with phenanthrene, fluoranthene, pyrene, and benzo[b]fluoranthene being the most abundant. These were mainly attributed to industrial emissions, thermal power generation, and wastewater discharge. Similarly, Semenov et al. [76] measured 0.050–0.700 mg/kg of PAHs in Lake Baikal, Russia. The profile was dominated by high molecular weight compounds with 4–6 rings, with benzo[b]fluoranthene (BbF) being the most abundant, mainly linked to biomass and fossil fuel burning. In Algeria’s Soummam River, PAH levels ranged from 1.86 to 22.94 mg/kg and were dominated by benzo[ghi]perylene, chrysene, phenanthrene, and pyrene. Carcinogenic PAHs accounted for 16.8–56.2% of the total and were mainly derived from petroleum, vehicle emissions, and biomass burning [72]. In the Barents and Norwegian Seas, Koltovskaya and Nemirovskaya [73] reported PAHs concentrations of 0.002–17.000 mg/kg. These included low molecular weight compounds, such as naphthalene and phenanthrene, and high molecular weight compounds, like fluoranthene, pyrene, and benzo[a]pyrene. The contamination was mainly petrogenic from natural hydrocarbon inputs. Likewise, Reznikov et al. [6] reported 0.0087–0.6708 mg/kg in Lake Baikal sediments, Russia, mainly of petrogenic origin and influenced by long-term wastewater and riverine inputs.

## 4. Fate and Transformation Mechanisms of Pollutants in Bottom Sediments

The fate of contaminants in bottom sediments is controlled by physical, chemical, and biological processes shaped by grain size, redox potential, organic matter, and microbial activity. These mechanisms prescription whether pollutants remain bound, transform into more mobile or toxic forms, or degrade into less harmful products, with pathways differing across nutrients, HMs, MPs, and organic micropollutants.

### 4.1. Physical Transport Mechanisms: Sedimentation

Sedimentation represents the primary physical pathway by which pollutants are transferred from the water column to bottom sediments through gravitational settling of particulate matter [17,51,76,77]. Most pollutants are not deposited in a freely dissolved form. Instead, they are generally transported while attached to suspended solids such as particulate organic matter (POM), mineral grains like clays and silts, or aggregated flocs through physical attachment [6,74,77]. The efficiency of sedimentation can depend on particle characteristics such as size, density, shape, and aggregation. These factors together determine the settling velocity [1,78]. According to Stokes’ law, particles with larger sizes and higher density settle faster. In contrast, fine or low-density particles may remain in suspension for a longer period, allowing them to move horizontally before settling [8,78].

Sedimentation is strongly affected by hydrodynamic conditions. Calm water generally allows particles to settle, while turbulence or fast-flowing water can delay settling or resuspend fine particles [1,17]. Conversely, moderate turbulence may help particles collide and aggregate, which can increase the rate of sedimentation. Therefore, the balance between deposition and resuspension depends largely on how fast particles settle compared to the shear stress on the sediment bed [78,79]. Extreme events such as floods and storms tend to increase sediment movement, carrying contaminated particles into the water column or onto nearby land [17,25,80].

The sedimentation of different pollutants may depend on the carrier phase to which they are bound. For example, when nutrients like phosphorus and nitrogen bind to POM or form flocs, their transport to the sediment can be increased [15,38]. HMs are mainly attached to fine mineral particles and organic substrates with a large surface area, which likely supports their stable deposition in sediments [25,81]. The behavior of MPs might depend on their density, shape, and surface properties. Some particles sink quickly, while others float or become resuspended. Over time, colonization by biofilms can increases their density and helps them settle [8,63]. Pesticides and PhACs are mainly transported by fine sediments and organic-rich particles, so their sedimentation may depend on how these carrier particles settle [11,82]. PAHs are highly hydrophobic and bind to organic and carbon-rich particles, which helps them settle into benthic sediments [74,75]. Through these processes, sedimentation determines where and how particles with pollutants settle at the sediment–water interface. This placement may influence the chemical and biological reactions that shape their long-term fate in the aquatic environment.

### 4.2. Redox-Driven Speciation and Transformation

Steep redox gradients in water-associated bottom sediments can drive electron-transfer reactions that may influence the speciation, mobility, and transformation of HMs, nutrients, MPs, and organic contaminants. Redox gradients in sediments also appear to regulate the transformation and persistence of pesticides, PhACs, and PAHs [28,32,83]. In oxic sediments, PhACs, reactive chlorinated pesticides, and some PAHs might undergo oxidation on Fe(III) and Mn(IV) oxides or via microbial redox enzymes, forming hydroxylated or partially dechlorinated metabolites: R–H + MnO_2_ → R–OH + Mn^2+^; R–H + Fe(III)–oxide → R–OH + Fe^2+^ [84,85]. Under anoxic conditions, halogenated PhACs and pesticides can be reductively dehalogenated by sediment-bound electron donors: R–Cl + 2e^−^ + H^+^ → R–H + Cl^−^ [28,82,85]. PAHs resist direct reduction but may undergo one-electron oxidation on Fe(III)-enriched surfaces to radical cations (PAH•^+^), which could react with water or oxygen to yield hydroxylated products:

PAH•^+^ + H_2_O → PAH–OH + H^+^; PAH•^+^ + O_2_ → PAH–O_2_ [32,33,86]. In sulfidic sediments, metal sulfides (e.g., FeS, CuS) can catalyze reductive transformation of halogenated organics. Humic substances and quinone/semiquinone moieties can mediate long-range electron transfer between microbial donors and sediment-bound acceptors, enhancing both oxidative and reductive processes [32,83,85]. Transition metals, particularly Fe(III) and Cu(II), may promote environmentally persistent free radicals (EPFR) formation, modifying adsorbed organics by introducing carbonyl, quinone, or hydroxyl groups [33,84,85,86]. Overall, dynamic shifts between oxic, anoxic, and sulfidic conditions likely help regulate these redox-driven transformations, thereby determining contaminant speciation, reactivity, and long-term fate in sediments.

The HMs in bottom sediments, including Pb, Hg, Cd, As, Cr, Cu, and Zn, undergo redox-sensitive transformations that may influence their mobility and retention [24,25,27]. Under oxic conditions, these metals are immobilized via adsorption onto ferric hydroxides [Fe(OH)_3_] and manganese oxides [MnO_2_], or can precipitate as carbonates (PbCO_3_, CdCO_3_) and phosphates (Pb_3_(PO_4_)_2_) [24,27]. When sediments become anoxic, reductive dissolution of Fe(III) and Mn(IV) oxides may releases metals into porewaters according to: M–FeOOH(s) + e^−^ → M^2+^ + Fe^2+^ + OH^−^ (where M = Pb^2+^, Cd^2+^, Zn^2+^, Cu^2+^, Hg^2+^, Cr^3+^) [24,25,26,27]. The liberated metals could diffuse toward the sediment–water interface or react with biogenic sulfide to form highly insoluble sulfide minerals, such as galena (PbS), greenockite (CdS), cinnabar (HgS), and covellite (CuS), via M^2+^ + H_2_S → MS(s) + 2H^+^ [24,25,27]. This binding is governed by stability; metastable phases (e.g., mackinawite, greigite) are highly reactive, posing a significant remobilization risk under transient oxic events, unlike the more stable phase, pyrite (FeS_2_) [24,25,26,27]. Mercury is partially reduced to volatile elemental Hg^0^ under reducing conditions. This may facilitate atmospheric release (Hg^2+^ + 2e^−^ → Hg^0^) [24,27]. The As and Cr can show distinctive redox behavior: arsenate [As(V)] is reduced to the more mobile and toxic arsenite [As(III)] (HAsO_4_^2−^ + 2e^−^ + 2H^+^ → H_3_AsO_3_), whereas hexavalent chromium (Cr(VI)) is reduced to trivalent Cr(III) (CrO_4_^2−^ + 3e^−^ + 4H^+^ → Cr^3+^ + 2H_2_O), which can precipitate as hydroxides or adsorb onto Fe/Mn oxides, stabilizing it in sediments [25,26,27,87]. Environmental factors such as pH, redox potential, sulfide levels, and organic matter strongly affect these processes. They likely help determine whether metals remain sequestered in sediments or become bioavailable in the surrounding water [25,26,27,53,88]. As a result, changes in redox conditions can control adsorption, precipitation, dissolution, and sulfide-driven sequestration, which together influence the long-term fate of HMs in sediment–water systems [24,26,27].

In bottom sediments, MPs are transformed by oxidative reactions that may depend on reactive oxygen species (ROS) and repeated cycles of quinone activity [30,89]. In oxic layers, ROS such as hydroxyl radicals (·OH), superoxide (O_2_^−^·), and singlet oxygen (^1^O_2_) are generated through photolysis of humic substances, microbial metabolism, and surface Fe^2+^/Fe^3+^ cycling [30,31]. These ROS can remove hydrogen atoms from polymer chains (–C–H + ·OH → –C· + H_2_O), forming carbon-centered radicals that react with oxygen to produce peroxyl radicals (–C· + O_2_ → –C–OO·) [30,31,90]. Decomposition of hydroperoxides generates alkoxyl radicals (–C–OOH → –C–O· + ·OH), which may drive chain scission and form carbonyl and carboxyl groups [31,89,90]. In aromatic polymers, resonance-stabilized radicals can form EPFRs, such as semiquinone and phenoxy radicals, which might reversibly exchange electrons with oxygen (–C_6_H_4_O· + O_2_ ⇌ –C_6_H_4_=O + O_2_^−^·) [30,89,91]. Strong oxidants such as H_2_O_2_ and O_3_ further convert hydroxylated moieties to quinones (–C–OH + [O] → –C=O + H_2_O), enhancing redox potential and potentially enabling localized redox coupling in sediments [30,89]. Sediment-derived H_2_O_2_ further oxidizes hydroxylated moieties to quinones (–C–OH + H_2_O_2_ → –C=O + H_2_O), enhancing localized redox potential. Under suboxic or anoxic conditions, reductive dissolution of Fe/Mn may oxides changes porewater redox states, and microbial biofilms can reduce surface quinones to semiquinone radicals, sustaining radical cycling [30]. Together, these processes may contribute to oxidative fragmentation of MPs, formation of oxygenated functional groups, and persistence of radicals, potentially integrating microplastics into sediment redox dynamics.

Phosphorus and nitrogen undergo tightly coupled redox-driven transformations in bottom sediments, which may be governed by steep oxic–anoxic gradients [15,16,92]. Under oxic conditions, phosphate (PO_4_^3−^) is immobilized through adsorption or co-precipitation with Fe(III) and Al(III) hydroxides, and to a lesser extent with Mn(IV) oxides [92,93,94]. Reductive dissolution of these minerals under anoxia can releases PO_4_^3−^ into porewaters: Fe(OH)_3_(s) + e^−^ + 3H^+^ → Fe^2+^ + 3H_2_O [16,92,93]. In sulfidic zones, Fe^2+^ may forms FeS (Fe^2+^ + HS^−^ → FeS(s) + H^+^), preventing Fe(III) re-oxidation and likely sustaining PO_4_^3−^ release. In Fe-rich, low-sulfide environments, PO_4_^3−^ can stabilizes as vivianite [Fe_3_(PO_4_)_2_·8H_2_O] [93,95]. Alkalinity enhances desorption via OH^−^/PO_4_^3−^ exchange, and microbial mineralization of organic P may provide additional soluble PO_4_^3−^ [92,93]. Upon re-oxygenation, Fe^2+^ is re-oxidized and precipitates as Fe(OH)_3_ (4Fe^2+^ + O_2_ + 10H_2_O → 4Fe(OH)_3_(s) + 8H^+^), recapturing PO_4_^3−^ [16,93].

Nitrogen cycling begins with ammonification of organic N to NH_4_^+^, which can diffuse into oxic microzones where it undergoes nitrification (NH_4_^+^ + 2O_2_ → NO_3_^−^ + 2H^+^ + H_2_O) [95,96]. In anoxic layers, NO_3_^−^ reduction may proceed via denitrification to N_2_ (2NO_3_^−^ + 10e^−^ + 12H^+^ → N_2_ + 6H_2_O), or dissimilatory nitrate reduction to ammonium (DNRA), which conserves N as NH_4_^+^ (NO_3_^−^ + 8e^−^ + 10H^+^ → NH_4_^+^ + 3H_2_O) [16,95,97]. Anammox can removes fixed N through anaerobic oxidation of NH_4_^+^ with NO_2_^−^ (NH_4_^+^ + NO_2_^−^ → N_2_ + 2H_2_O), while Feammox might couples anaerobic NH_4_^+^ oxidation to Fe(III) reduction, potentially producing N_2_ or NO_2_^−^ and Fe^2+^ [93,96,97,98]. These pathways interact dynamically: re-oxygenation may restore Fe(III)-PO_4_^3−^ binding and stimulates nitrification, which in turn can supply substrates for denitrification and anammox [16,95,96]. Thus, redox oscillations likely dictate whether sediments act as long-term sinks through vivianite formation, Fe(III) reprecipitation, and N_2_ production, or as sources through FeS-mediated PO_4_^3−^ release and NH_4_^+^ retention via DNRA [16,95,97].

### 4.3. Sorption Dynamics and Environmental Factors

Sorption, through adsorption onto particle surfaces and absorption into sediment matrices, typically establishes the primary interactions between pollutants and sediments, forming the basis for subsequent mechanistic retention processes [8,11,38]. HMs are generally retained in sediments primarily through adsorption onto mineral surfaces, cation exchange, and complexation with organic matter [81]. Clays and humic substances provide abundant reactive sites, where divalent and trivalent metals such as Cd^2+^, Pb^2+^, Cu^2+^, and Fe^3+^ can form relatively stable surface complexes [25,81,99]. Carbonates (CaCO_3_, MgCO_3_) and metal hydroxides [e.g., Fe(OH)_3_, Al(OH)_3_] also may act as sorbents, particularly in sediments with higher buffering capacity [25]. The pH (controlled by H^+^ activity) strongly regulates sorption strength. Under acidic conditions, H^+^ ions may compete for binding sites, weakening metal attachment and promoting desorption, while near-neutral to slightly alkaline conditions enhance ≡M–OH surface complexation and cation exchange stability [88,99]. Ionic strength further modifies these equilibria by compressing the electrical double layer, reducing electrostatic attraction and favoring desorption in saline or ion-rich systems where competing cations such as Na^+^, Ca^2+^, Mg^2+^, and K^+^ are abundant [25,81]. Sediment texture and composition also influence retention capacity. Fine-grained, clay- and silt-rich sediments with high surface area exhibit stronger sorption compared to coarse or quartz (SiO_2_)-dominated fractions [13,25,81]. Similarly, total organic carbon (TOC) can enhance sorption by providing both polar functional groups (e.g., –COOH, –OH, –C=O) and nonpolar domains (hydrophobic moieties) for metal association [25,99]. The distribution of metals among sorbed fractions can be described by sorption-based partitioning: exchangeable, carbonate-bound, oxide-associated, organic-bound, and residual [99]. Their relative mobility is generally considered to follow the order: exchangeable > carbonate > oxide-bound > organic-bound > residual [25,99].

Nutrient and metal retention in sediments is governed by sorption equilibria that may involve mineral surfaces, organic matter, and ionic competition [15,16,100]. Orthophosphate (PO_4_^3−^) typically binds strongly to Fe and Al oxyhydroxides [e.g., FeOOH, Al(OH)_3_] through inner-sphere ligand exchange, with sorption capacity often enhanced by fine-grained minerals that provide large surface areas and by oxyhydroxide coatings on clays that supply abundant reactive sites [38,39,100]. Calcium carbonate (CaCO_3_) phases can further immobilize PO_4_^3−^ via surface precipitation and co-precipitation, particularly under alkaline conditions, whereas acidification can promote CaCO_3_ dissolution and PO_4_^3−^ release [38]. Ammonium (NH_4_^+^) is retained mainly through cation exchange with negatively charged clay interlayers, with reversibility governed by sediment cation exchange capacity; however, partial fixation in illite or vermiculite layers under reducing conditions may reduce its exchangeability. In contrast, nitrate (NO_3_^−^) remains highly mobile, interacting only weakly with mineral or organic functional groups [16,38]. Organic matter can contributes additional retention pathways by complexing PO_4_^3−^ with carboxyl (–COOH), hydroxyl (–OH), and phenolic (–Ar–OH) groups, and by providing exchange sites for NH_4_^+^, while also potentially influencing the stability of organic P fractions [16,38,100]. Environmental conditions regulate these sorption equilibria. pH modifies both surface charge and nutrient speciation: proton (H^+^) competition under acidic conditions weakens binding, while near-neutral to slightly alkaline conditions favor PO_4_^3−^–metal complexation and stable NH_4_^+^ exchange. However, excess alkalinity may destabilize sorption complexes and promote desorption [15,16]. Ionic strength (I) compresses the electrical double layer and can intensifies competition among cations (e.g., Na^+^, Ca^2+^, Mg^2+^) and anions (Cl^−^, SO_4_^2−^), thereby reducing electrostatic attraction and enhancing desorption in ion-rich systems. Temperature may influences sorption kinetics by accelerating surface reactions, while also shifting equilibria depending on whether reactions are endothermic or exothermic; nevertheless, the equilibrium distribution is generally controlled by mineralogy, organic matter, and ionic environment [34]. Collectively, these processes define the balance between nutrient immobilization and remobilization in sediments through sorption-based partitioning mechanisms [34,38,39].

The MPs act as low-affinity but mobile sorbents whose sorption dynamics may be governed by their polymer backbone chemistry, surface functional groups, and surrounding sediment matrix composition [8,101,102]. The hydrophobic hydrocarbon chains of polymers can drive partitioning with non-polar organic contaminants via van der Waals forces and hydrophobic interactions, while oxidation and hydrolysis reactions at the surface may generate functional groups (–COOH, –OH, –C=O), which can enable hydrogen bonding, electrostatic interactions, and π–π stacking with aromatic pollutants and transition metal ions [8,101]. Biofilm extracellular polymeric substances (EPS) introduce additional charged functional groups and polar moieties, modifying the surface zeta potential and may enhance heteroaggregation with clay minerals and organic particulates, thereby altering pollutant binding affinity [8]. These interactions are thermodynamically reversible and kinetically labile, with protonation-deprotonation equilibria of surface functional groups (–COOH, –OH) typically controlled by local pH, and ionic strength-dependent desorption can regulate remobilization of contaminants into sediment porewaters [8,101,103]. Polymer crystallinity, amorphous domains, and particle morphology likely dictate the density of accessible adsorption sites, with rubbery, high-surface-area polymers (e.g., PE) generally showing faster uptake compared to glassy polymers (e.g., PS), and fragmentation into smaller particles may increasing molecular surface contact and adsorption energy [8,101,102]. For amphiphilic contaminants such as PFAS, adsorption can occur via hydrophobic partitioning of perfluorinated tails coupled with electrostatic interactions of charged head groups, which may be modulated by the MP surface potential and local pH-dependent speciation [101,103]. Thus, MP–pollutant interactions represent dynamic chemical equilibria, likely governed by surface oxidation state, functional group chemistry, polymer molecular structure, and electrostatic forces, resulting in temporally labile yet chemically significant retention within sediments [8,101,102].

Organic pollutants in water, including PAHs, PhACs, and pesticides, are retained in bottom sediments through specific mechanistic interactions that may occur [11,74,82]. PAHs, being non-polar and aromatic, typically partition into sedimentary organic matter and condensed carbonaceous phases such as black carbon or soot via hydrophobic interactions, while π–π stacking with aromatic moieties in humic substances can further stabilize sorption [104,105]. Ionizable PhACs and pesticides may engage in electrostatic interactions with negatively charged clay or humic surfaces, where cationic forms (R–NH_3_^+^) bind directly and anionic forms (R–COO^−^) may be stabilized via cation bridging with divalent/trivalent ions (Ca^2+^, Mg^2+^, Fe^3+^) [11,68]. Polar functional groups, including –OH, –COOH, –C=O, –CONH_2_, and Ar–OH, can participate in hydrogen bonding with oxidized sediment surfaces [11,82]. Micropores in fine silt and clay fractions (2–50 nm) may provide size-selective sequestration, favoring small- to medium-size PAHs and low-molecular-weight pesticides, while steric hindrance limits larger molecules [74,82]. Surface complexation can further stabilize ionizable pollutants by forming inner- or outer-sphere complexes with sedimentary functional groups in the presence of metal cations [11,75]. Sediment weathering and particle fragmentation may expose additional reactive sites, enhancing hydrophobic, electrostatic, and hydrogen-bonding interactions [74,75]. Environmental factors such as pH, ionic strength, and competing dissolved organic matter may modulate sorption by altering protonation equilibria, compressing electrical double layers, or occupying binding sites [4,11]. Together, these processes likely govern the retention, mobility, and bioavailability of PAHs, PhACs, and pesticides in water-associated bottom sediments.

### 4.4. Microbial Biodegradation and Biotransformation

Microbial communities in benthic sediments strongly influence how pollutants behave, and may control their retention, transformation, and movement at the sediment–water interface. These communities play a key role in N and P transformations through oxic–anoxic microgradients [9,96,100]. Organic nitrogen is mineralized to ammonium via microbial ammonification (R–NH_2_ + H_2_O → NH_4_^+^ + CO_2_), which in oxic microzones is typically oxidized by ammonia- and nitrite-oxidizing bacteria to nitrite and nitrate (NH_4_^+^ + O_2_ → NH_2_OH → NO_2_^−^; NO_2_^−^ → NO_3_^−^), limiting direct ammonium diffusion into overlying water [9,96,106]. In anoxic microzones, heterotrophic denitrifiers sequentially may reduce nitrate to dinitrogen gas (NO_3_^−^ → NO_2_^−^ → NO → N_2_O → N_2_), while anammox bacteria can convert nitrite and ammonium directly to N_2_ (NO_2_^−^ + NH_4_^+^ → N_2_ + 2H_2_O), helping remove reactive nitrogen from porewater and potentially controlling flux to overlying water [9,16,96]. Minor pathways such as DNRA (NO_3_^−^ + 2e^−^ + 2H^+^ → NH_4_^+^ + H_2_O) may recycle nitrogen under high organic carbon conditions [9,95,97]. Organic phosphorus is liberated from ester-bound compounds by microbial phosphatases (R–O–PO_3_^2−^ + H_2_O → ROH + H_2_PO_4_^−^), supplying potentially bioavailable phosphate for microbial assimilation and enzymatic turnover; polyphosphate accumulation and hydrolysis by specialized microbes may further modulate phosphorus availability [92,100]. These microbial transformations are likely governed by redox conditions, oxygen penetration, organic carbon content, and sediment micro-niches, controlling nutrient retention, speciation, and the flux of nitrogen and phosphorus between sediments and overlying water [9,16,95,96].

Microbial communities in bottom sediments mediate the transformation and retention of HMs at the sediment–water interface. In oxic microzones near the sediment surface, microbial activity can modulate local redox and pH, indirectly stabilizing Pb^2+^, Cd^2+^, Zn^2+^, and Cu^2+^, while arsenate [As(V)] may adsorbs to microbial surfaces, potentially limiting mobility into overlying water [24,27]. As sediments become anoxic, sulfate-reducing and methanogenic microbes generate sulfide (H_2_S) (SO_4_^2−^ + 8e^−^ + 10H^+^ → H_2_S + 4H_2_O), which reacts with dissolved and porewater metals to form insoluble sulfides (M^2+^ + H_2_S → MS(s) + 2H^+^), likely reducing metal flux to the water column [24,53]. Concurrently, arsenate is microbially reduced to arsenite [As(III)] (HAsO_4_^2−^ + 2e^−^ + 2H^+^ → H_3_AsO_3_) and can form thioarsenates under sulfidic conditions, which may modulate solubility and diffusion into overlying water [24,26]. Mercury can undergo microbial methylation by hgcAB-bearing microbes to methylmercury (MeHg), while demethylation and reduction processes may release Hg^2+^ or volatile Hg^0^ (Hg^2+^ + 2e^−^ → Hg^0^), controlling its potential mobilization into the water column [24,26,53,107]. Through these microbial-mediated transformations, HMs likely experience dynamic cycles of immobilization, release, sulfide precipitation, and biotransformation, ultimately influencing their bioavailability and flux between sediments and overlying water.

In water-associated bottom sediments, microbial biofilms can colonize MPs surfaces and secrete extracellular oxidative enzymes, generating ROS that can initiate polymer chain degradation [7,8,30,108]. Hydrogen abstraction from aliphatic C–H bonds (–CH– + ·OH → –C· + H_2_O) produces carbon-centered radicals (–C·), which react with molecular oxygen to form peroxyl radicals (–C· + O_2_ → –C–OO·) [7,108,109]. Subsequent enzymatic oxidation and hydrolysis may cleave polymer units and labile bonds (–CH_2_– + O → –CH–OH; –CO–O– → –COOH + –OH), yielding smaller fragments accessible to radical attack [7,8,108]. Homolytic decomposition of peroxyl radicals (–C–OOH → –C–O· + ·OH) can drive chain scission and introduces oxygen-containing functional groups such as hydroxyl (–OH), carbonyl (–C=O), and carboxyl (–COOH) [108,109]. Within biofilm microenvironments, redox-active moieties may undergo reversible single-electron transfer (Q + e^−^ ⇌ Q·^−^), sustaining ROS regeneration and potentially enabling continuous oxidative degradation [30,109,110]. Oxic microzones typically maintain persistent ROS flux and polymer oxidation, whereas anoxic niches may facilitate reductive regeneration of quinone-like radicals, which may maintain localized redox cycling [30,89,109,110]. Progressive polymer fragmentation and functionalization can produce redox-active sites that interact with Fe(III)/Mn(IV) oxides and sedimentary organic matter, integrating microplastics into redox-mediated transformation networks without necessarily favoring specific microbial taxa [8,109,110].

In oxic sediment layers, microbial enzymatic processes can drive the oxidative transformation of PhACs, PAHs, and pesticides [11,28,111]. PhACs typically undergo hydroxylation catalyzed by cytochrome P450 monooxygenases, producing more polar metabolites (R–H + O_2_ + NAD(P)H → R–OH + H_2_O + NAD(P)^+^) [11,112]. PAHs are generally dioxygenated at aromatic rings by microbial dioxygenases to form dihydrodiols, which are subsequently cleaved into carboxylated intermediates (C_6_H_4_–C_6_H_4_ + O_2_ + NADH → C_6_H_4_(OH)–C_6_H_4_(OH) → carboxylates) [29,84,111,113]. Ester- and amide-containing pesticides may be hydrolyzed by microbial esterases and amidases, generating smaller, soluble fragments (R–COOR′ + H_2_O → R–COOH + R′–OH) [28,114,115]. These oxidative and hydrolytic modifications can increase solubility and potentially enhance bioavailability, enabling stepwise mineralization to CO_2_ and H_2_O [11,29,115].

In anoxic sediment layers, reductive transformations typically predominate. PhACs and nitroaromatic pesticides are enzymatically reduced via dehalogenation and nitro-group reduction using electron donors derived from fermentative processes (R–Cl + 2e^−^ + H^+^ → R–H + Cl^−^; Ar–NO_2_ + 6e^−^ + 6H^+^ → Ar–NH_2_ + 2H_2_O) [28,115,116]. PAHs are partially hydrogenated and destabilized, which may facilitate downstream fermentative breakdown [117]. Complex organics from all classes are further converted via fermentative and syntrophic processes into short-chain acids, alcohols, and H_2_, which are terminally oxidized by anaerobic pathways to CH_4_, CO_2_, or H_2_S [115,117]. Under fluctuating redox conditions, facultative microbial processes may enable cometabolic transformations [115,116,117]. Xenobiotics from PhACs, PAHs, and pesticides are co-transformed alongside primary substrates, generating partially oxidized or reduced intermediates [29,116,117]. Oxidative coupling of hydroxylated intermediates can lead to polymerization into humic-like matrices, forming non-extractable bound residues [116,118]. Collectively, these oxic and anoxic microbial processes govern the stepwise bioconversion of PhACs, PAHs, and pesticides, directing either potential complete mineralization or long-term sequestration within sediment matrices.

### 4.5. Synergistic and Antagonistic Interactions Among Pollutants

#### 4.5.1. Microplastics as Vectors and Modulators of Pollutants

MPs modulate contaminant behavior in sediments through well-defined physicochemical mechanisms [119,120]. Hydrophobic partitioning into polymer matrices, π–π stacking between aromatic moieties, and electrostatic interactions with surface functional groups can govern sorption of both organic and inorganic pollutants [20,120,121]. These processes may prolong the persistence and transport of hydrophobic organics, such as PAHs and pesticides, by retaining them on particle- or biofilm-modified surfaces, which potentially reduce immediate degradation and may facilitate redistribution through resuspension [120,121]. Sorption of cationic metals (Pb^2+^, Cd^2+^, Cu^2+^, Zn^2+^) and ionizable pharmaceuticals onto oxidized or biofilm-coated MPs can decrease their freely dissolved concentrations, producing an apparent antagonistic effect relative to direct mineral sorption [20,120]. With aging and biofilm colonization, polymer surfaces may become more oxidized and porous, enhancing desorption and pollutant release into porewaters, which potentially reverse initial immobilization [30,89,110,119]. In addition, microbial biofilms established on MPs can modify local redox microenvironments, influencing electron transfer processes and thereby potentially regulating contaminant exchange at the sediment–water interface [20,89].

#### 4.5.2. Nutrient-Driven Modulation of Pollutant Fate

Nitrogen and phosphorus strongly interact with other contaminants through their control over redox dynamics and mineral stability [22,122,123]. Oxygen depletion caused by nutrient enrichment can accelerate reductive dissolution of Fe and Mn oxides (Fe(OH)_3_(s) + e^−^ + 3H^+^ → Fe^2+^ + 3H_2_O), releasing sorbed metals (Cd^2+^, Zn^2+^, Pb^2+^) and phosphate into porewaters, which may establish positive feedback between eutrophication and contaminant mobilization [21,23,124]. Accumulated ammonium fuels DNRA and Feammox pathways (NO_3_^−^ + 8e^−^ + 10H^+^ → NH_4_^+^ + 3H_2_O), producing Fe^2+^ that can destabilize oxide phases and potentially promote additional release of bound pollutants [123]. In contrast, nutrient-driven organic matter deposition may enhance sulfide generation under anoxic conditions, which immobilizes metals as highly insoluble sulfides (M^2+^ + H_2_S → MS(s) + 2H^+^, where M = Pb, Cd, Hg) [23,124]. Phosphate may compete with anionic pesticides (e.g., triazines) and ionizable pharmaceuticals (e.g., sulfonamides) for sorption onto Fe/Al oxide surfaces, displacing them into porewaters, whereas strong phosphate binding to these mineral phases can also indirectly stabilize other anionic species by limiting exchange capacity [21,122]. Thus, nutrient enrichment can produce both synergistic outcomes, where mobilization of pesticides, pharmaceuticals, and metals is enhanced, and antagonistic outcomes, where immobilization is favored, depending on mineralogy, organic matter flux, and prevailing redox conditions [22,122,123].

#### 4.5.3. Heavy Metal–Organic Contaminant Coupling Mechanisms

HMs interact with pesticides, pharmaceuticals, and PAHs through catalytic, inhibitory, and complexation pathways that can modify transformation and mobility [20,22,125]. Transition metals such as Cu^2+^, Fe^3+^, and Mn^4+^ catalyze Fenton-like reactions (M^n+^ + H_2_O_2_ → M^n−1^ + ·OH + OH^−^), generating hydroxyl radicals that may accelerate oxidative degradation of PAHs and pesticides [126,127]. In contrast, non-redox-active metals such as Cd^2+^ and Pb^2+^ bind to sulfhydryl groups in microbial enzymes, which can inhibit the biodegradation of pesticides and pharmaceuticals, potentially prolonging their persistence [127,128]. Complexation of pharmaceuticals with Cu^2+^ or Fe^3+^ may alter their sorption affinity and slow desorption kinetics, while interactions of Hg^2+^ and As species with natural organic matter produce organometallic derivatives such as methylmercury (Hg^2+^ + CH_3_^−^ → CH_3_Hg^+^) and thioarsenates that may exhibit higher toxicity and mobility [20,125,129]. Co-exposure to metals and organics can generate synergistic oxidative stress, as when Cu^2+^ amplifies ROS formation in the presence of PAHs, whereas antagonistic effects may occur when Cd^2+^ inhibits enzymatic degradation of pesticides [20,22,125]. The long-term co-occurrence of metals and pharmaceuticals can apply selective pressure on sediment microbial communities, encouraging resistance traits that might indirectly reduce the efficiency of biodegradation [22,128]. Overall, these mechanisms show that interactions between metals and organic compounds can either increase transformation or decrease pollutant degradation, depending on redox conditions, complexation balances, and microbial tolerance levels.

## 5. Environmental Impacts of Pollutants in Sediments

**Figure 3:**
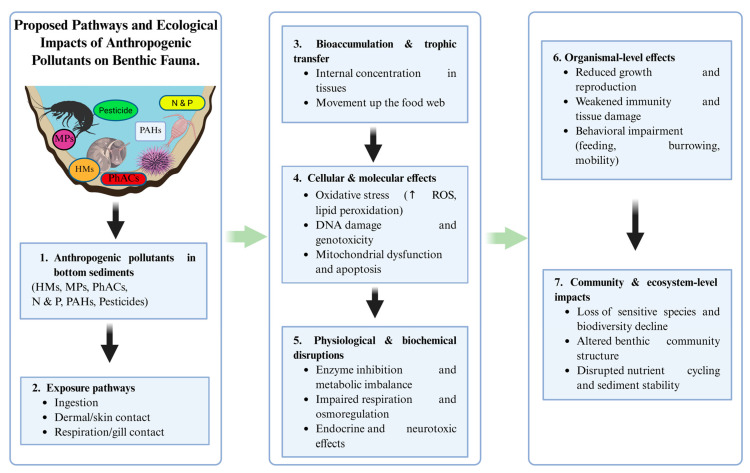
Proposed toxicological pathway and ecological impacts of anthropogenic pollutants in bottom sediments on benthic fauna. Created in BioRender. Maqsood, A. (2025) https://BioRender.com/bmfadwa (accessed on 15 October 2025).

**Figure 4:**
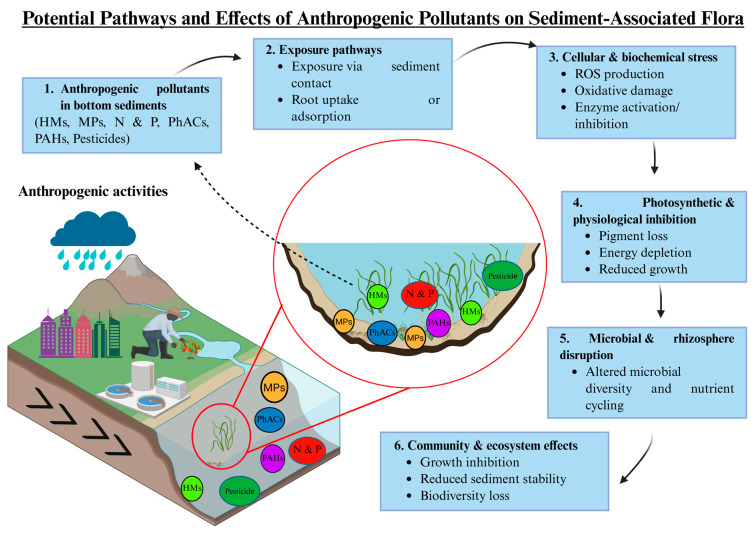
Mechanistic pathway showing the potential effects of anthropogenic pollutants on sediment-associated flora. Created in BioRender. Maqsood, A. (2025) https://BioRender.com/4dtx7fq (accessed on 15 October 2025).

Pollutants in bottom sediments can accumulate in aquatic organisms, potentially harm bottom-dwelling species and plants, may move through food webs, and might pose risks to both ecosystems and human health [17,18], as summarized in Figure 3 and Figure 4. These contaminants include nutrients, pesticides, HMs, PhACs, PAHs, and MPs. Through their interactions with living communities, they can influence key ecosystem processes and may determine how long pollutants persist and how strongly they impact the environment.

### 5.1. Toxicological Effects on Benthic Fauna

Benthic fauna mainly consist of invertebrates like worms, crustaceans, mollusks, and insect larvae that live within sediments [75,130]. They play important roles in nutrient cycling, maintaining sediment structure, and forming a key part of aquatic food webs. These invertebrates can interact directly with pollutants in the sediments through feeding, skin contact, and breathing [17,25]. Contact with these potentially toxic substances may lead to both acute and chronic health effects [19], as summarized in Figure 3. Invertebrates that accumulate HMs can suffer disruptions in enzymatic activity and cell functions, leading to oxidative stress, DNA damage, and genotoxic effects [17]. In crustaceans, HMs exposure may also disrupt metabolism, breathing, and sperm quality, particularly under longer or stronger exposure [17,131]. Such effects can disturb normal nervous and physiological functions, potentially causing developmental defects, reduced growth and reproduction, weakened immunity, and increased mortality [131]. Supporting these observations, Demina et al. [18] reported HMs concentrations in Arctic marine invertebrates: in the intestinal tissues of *Myriotrochus rinkii,* Pb, Ni, Cu, Zn, As, and Cd were 6.8, 15, 13.3, 38, 11, and 0.54 mg/kg, respectively; the isopod *Saduria sibirica* had whole-body levels of As, Zn, Cu, and Cd at 15.3, 73, 92, and 3.58 mg/kg, respectively; and soft tissues of *Portlandia arctica* contained Cd, Zn, Ni, As, Cu, and Pb at 5.2, 89, 21.1, 9.4, 7.6, and 2.13 mg/kg, respectively.

Pesticides with lipophilic properties can readily penetrate the cell membranes of sediment-dwelling invertebrates, leading to oxidative stress through the potentially excessive generation of ROS [80]. This can result in lipid peroxidation and depletion of intracellular antioxidants such as glutathione, disrupting enzymatic activity and cellular homeostasis. Consequently, these molecular disturbances may trigger apoptosis and cell death [80,132]. In response, defense mechanisms including the expression of heat shock proteins and activation of stress-related signaling pathways (e.g., p53, SAPKs) are initiated; however, under high pesticide loads, these protective systems might become overwhelmed and less effective [80,133]. Supporting this, Primost et al. [130] reported significant organochlorine pesticide contamination, particularly endosulfan, p,p’-DDE, and endrin, in benthic invertebrates including bivalves, gastropods, polychaetes, crabs, and sea urchins collected from the Atlantic Patagonian harbor, with concentrations reaching up to 0.022 mg/kg. Sediment-bound PAHs can induce oxidative stress and DNA damage in bivalves and polychaetes, compromising vital physiological functions like respiration and filtration [134,135]. In *Marphysa sanguinea*, PAH concentrations reached up to 6.02887 mg/kg, leading to elevated immune biomarkers (COX and lysozyme), depletion of energy reserves (triacylglycerol), and alterations in membrane lipid composition, reflecting significant metabolic and physiological impairment likely associated with sediment contamination [134]. Furthermore, Primost et al. [130] reported PAH up to 0.028 mg/kg in bivalves, 0.27 mg/kg in gastropods, 0.967 mg/kg in polychaetes, and 8.5 mg/kg in sea urchins, highlighting the potential for significant bioaccumulation among benthic invertebrates.

MPs negatively affect benthic invertebrates such as polychaetes, amphipods, copepods, and bivalves including *Mytilus galloprovincialis* and *Crassostrea gigas* [63,136]. These organisms often ingest MPs because they resemble sediment particles, which can cause physical damage to the gut lining and may impair nutrient absorption. Exposure to MPs can induce oxidative stress, lipid peroxidation, DNA damage, and mitochondrial dysfunction, which may trigger apoptosis (programmed cell death) [137,138,139]. MPs also may disrupt osmoregulation and weaken the immune system, contributing to reduced growth, reproductive disorders, and increased mortality [19,138]. Additionally, adherence of MPs to appendages such as antennae and feeding legs can hamper normal movement and feeding behaviors [19]. Reproductive impairments may include altered gametogenesis, larval deformities, and modulation of genes involved in detoxification and immune responses [63,137,138]. According to Trestrail et al. [136], eating spherical MPs decreased digestive enzymes in *Mytilus galloprovincialis,* which may make it harder to process food, potentially weakening energy reserves and limiting growth and survival. It is worth emphasizing that MP has been found in all marine organisms, i.e., it has been detected in the digestive tract of fish, dolphins, humpback whales and seabirds, as well as in the soft tissues of mussels.

PhACs can harm benthic invertebrates by affecting their behavior, reproduction, and development [140]. These compounds can cause oxidative stress, neurotoxic, and genotoxic effects, which may damage DNA and cell functions [141]. Endocrine disruption and weakened immunity may further decrease survival and overall fitness, collectively potentially destabilizing sediment-dwelling communities [140,141]. For example, in the Ganjiang River, China, viviparid snails (*Cipangopaludina chinensis*) were found to accumulate antibiotics at concentrations of 0.94–1.03 mg/kg [77]. Excess nitrogen and phosphorus can also indirectly affect benthic invertebrates by changing sediment chemistry and decreasing oxygen levels [35,142]. Excess nutrients may cause eutrophication, which leads to algal blooms that decay and create low-oxygen (hypoxic or anoxic) conditions, potentially increasing oxidative stress and ROS production [35]. These stressors may cause DNA damage, mitochondrial dysfunction, and cell death (apoptosis) in sensitive species. Higher ammonia and nitrite levels can disturb osmoregulation and weaken immunity, leading to reduced growth, reproduction, and survival [35,142,143]. Collectively, these effects may harm the physiological health of organisms and weaken the stability of benthic communities.

### 5.2. Effects on Sediment-Associated Flora

Submerged macrophytes, emergent plants, and microalgae in sediments are important because they help stabilize the sediment, supply oxygen, and provide habitats for aquatic life [144,145]. Among these plants, *Vallisneria natans* is considered sensitive to HMs when levels exceed their tolerance limit. Such exposure can damage plant structure, reduce chlorophyll, disrupt photosynthesis, and decrease growth and biomass [146]. Cd and Pb can also cause oxidative stress by increasing ROS, which may damage cells and activate programmed cell death. In response, the plant may increase the activity of antioxidant enzymes such as superoxide dismutase (SOD), peroxidase (POD), and catalase (CAT) to reduce this stress [147]. Over time, continuous exposure may reduce the protective effect of these enzymes, leading to higher malondialdehyde (MDA) levels, which can damage cell membranes through lipid peroxidation. Continuous metal stress may cause genetic mutations and damage chromosomes, disrupting normal cell division and plant growth [146,147].

In addition to HMs, sediment-associated plants also face significant challenges from MPs and nutrient pollution. Lin et al. [148] reported that MPs in sediments negatively affect *Vallisneria denseserrulata.* At 1000 mg/kg MPs, plant height was reduced by 19–22% and biomass by 11–16%. MPs also appeared to alter rhizosphere microbial communities, lowering diversity and potentially suppressing carbon and nitrogen cycling. Specifically, phototrophy, nitrogen fixation, and nitrate reduction were reported to be suppressed by up to 68%, 24%, and 18%, respectively. Similarly, exposure of *Vallisneria natans* to MPs (10–50 mg/L) can induce oxidative stress, elevate antioxidant enzyme activities (SOD, POD, CAT, glutathione S-transferase), and cause cellular damage including organelle disruption, while possibly altering microbial biofilm communities on leaves [149]. In Negombo Lagoon, Sri Lanka, Rathnayake et al. [150] observed MPs abundance of 5000–6000 particles/kg on leaves and roots of the seagrass *Halodule pinifolia*. Similarly, in the Venice Lagoon, Adriatic Sea (Italy), Sfriso et al. [151] found MPs contamination in benthic macroalgae (seaweeds) ranging from 160 to 330,000 particles/kg.

Nutrient enrichment also may induce considerable stress. In planted microcosm experiments, high levels of COD, TN, NH_4_^+^-N, and TP were observed to cause oxidative stress in *Vallisneria natans* (submerged), *Nymphaea* spp. (floating-leaved), and *Lythrum salicaria* (emergent). Notably, *Vallisneria natans* showed signs of elevated MDA, suppressed antioxidant enzyme activity, and reduced chlorophyll content, leading to visible leaf wilting and necrosis. The inhibitory effects at high pollutant levels (COD ≥ 115 mg/L, TN ≥ 20 mg/L, NH_4_^+^-N ≥ 18 mg/L, TP ≥ 4.5 mg/L) appeared to overwhelm physiological regulation, resulting in lower phytoremediation capacity and plant decline [144]. Moreover, high sediment nitrogen and phosphorus, especially at an N-to-P ratio near 40:1, may suppress the growth of benthic *Cladophora* algae by promoting dense mats of benthic cyanobacteria that outcompete algae for light and nutrients, resulting in a significant decline in algal biomass [145]. Collectively, these pollutants can impact plant health, microbial interactions, and ecosystem functions in sediment-associated flora.

Organic contaminants, particularly pesticides, can disrupt redox homeostasis in aquatic plants by generating ROS, which may impair vital cellular functions. In response, submerged macrophyte plants activate SOD, CAT, POD, along with stress-related signaling pathways like MAPK and PI3K, to potentially help minimize oxidative damage [80,132]. Among such contaminants, phosphites, commonly used as fungicides or biostimulants, have been shown to reduce leaf and root length, suppress growth, and decrease chlorophyll content in *Vallisneria natans*, primarily by inducing oxidative stress, as indicated by elevated levels of SOD and MDA [152]. Similarly, ciprofloxacin, a widely detected PhAC compound, caused significant physiological and biochemical stress in *Vallisneria natans*, including growth inhibition, pigment degradation, oxidative damage, and disruption of protein metabolism [153]. In natural environments, Qiu et al. [77] examined antibiotic accumulation in submerged plants from the Ganjiang River, China, including black algae (*Hydrilla verticillata*), bitter grass (*Vallisneria natans*), and foxtail algae (*Myriophyllum verticillatum*), with concentrations ranging from approximately 0.15 to 0.41 mg/kg, and black algae consistently appearing to have the highest levels. Additionally, a pot experiment assessed the effects of PAHs (phenanthrene and pyrene) on the salt marsh plant *Spartina alterniflora* in contaminated sediments. Exposure to these PAHs was observed to alter the rhizosphere microbial community, significantly reduce microbial biomass, and inhibit enzymatic activities such as dehydrogenase and polyphenol oxidase. This likely reduced nutrient cycling, lowered phytoremediation efficiency, and reduced plant growth and health [111]. Overall, pollutants such as HMs, MPs, nutrients, pesticides, PhACs, and PAHs can disrupt plant physiology, enzyme defense, and plant–microbe interactions, potentially weakening sediment-associated flora and the ecosystem services they provide.

### 5.3. Bioaccumulation and Biomagnification in Aquatic Food Webs

Pollutants in bottom sediments may move into aquatic food webs through bioaccumulation and biomagnification, potentially leading to serious ecological and health impacts [154]. Bioaccumulation is the process by which aquatic organisms take in and store contaminants from water, sediment, or food over time. Biomagnification refers to the tendency of these pollutants to become more concentrated at higher trophic levels as they are passed along the food chain [17,134]. In fish, exposure to HMs can cause neurotoxicity, which may damage both the central and peripheral nervous systems, suppress blood formation, impair heart function, harm organs, and increase mortality [154]. Waterfowl feeding on contaminated fish or invertebrates often experience immunological suppression, developmental anomalies, and impaired reproductive performance, including eggshell thinning and reduced hatchling viability [155]. Similarly, prolonged HMs exposure can threaten aquatic predators such as sea bass, dolphins, and otters by weakening immune responses and potentially causing neurological, kidney, and liver dysfunction [156]. The HMs accumulated through aquatic food may damage vital human organs, including kidneys, liver, brain, heart, and skin, leading to organ failure, neurological disorders, reproductive problems, cancer, or death [17]. These metals accumulate differently in specific organs and species; for instance, in fish, high concentrations were observed in the scales of Sperata seenghala for Cd (41.7 mg/kg), liver of *Catla catla* for Cu (21.5 mg/kg), gills of *S. seenghala* for Cr (15.3 mg/kg), and scales of *Cirrhinus cirrhosus* for Pb (15.3 mg/kg). The lowest levels were mostly found in the brains of *Hypophthalmichthys molitrix* and *C. cirrhosus*. In birds, *Anas clypeata* (Shoveler) appeared to show especially high Cd (66.6 mg/kg) and Cu (23.56 mg/kg) in kidneys and Pb (4.51 mg/kg) in lungs, while the lowest concentrations were generally observed in the feathers of *Aythya ferina* (Pochard) and the brain of *Mareca strepera* (Gadwall) [157].

In addition to HMs, PhACs and pesticides may pose significant ecological risks through bioaccumulation and toxicity in aquatic organisms and their predators. Castaño-Ortiz et al. [158] detected various PhACs, including analgesics, anti-inflammatories, and psychiatric drugs, in fish from the Ebro River Delta (Spain), with notable accumulation in fish liver (up to 0.166 mg/kg), plasma (up to 63 ng/mL), and muscle (up to 0.031 mg/kg). Similarly, exposure to CBZ at concentrations of 1, 10, and 100 mg/L appeared to induce oxidative stress, DNA damage, and cell apoptosis in Chinese rare minnows (*Gobiocypris rarus*) via activation of the Ras/Raf/ERK/p53 pathway, suggesting genotoxic and cytotoxic effects [159]. In higher predators, such as the African penguin (*Spheniscus demersus*), PhACs have been reported to disrupt hormonal balance, reduce fertility, impair reproductive behavior, and damage reproductive organs, potentially threatening population viability [160]. Similarly, bottlenose dolphins (*Tursiops truncatus*) may experience immunosuppression, increased infection risk, developmental toxicity, reproductive disorders, and higher mortality due to pharmaceutical contamination, with chronic exposure possibly contributing to physiological dysfunction and sublethal effects [161]. In fish, pesticides, particularly pyrethroids, can induce lipid peroxidation and oxidative stress, which may activate apoptotic signaling pathways such as p53 and JNK. These biochemical disruptions could impair cellular function, leading to cell death, and might negatively affect growth, reproduction, and survival, ultimately destabilizing the aquatic food web [80,133]. In bottlenose dolphins from the Gulf of Guayaquil, exposure to organochlorine pesticides, comprising up to ~50% of ΣPOP with p,p′-DDE concentrations as high as ~7.0 mg/kg lipid weight, has been associated with immune suppression and endocrine disruption, including anti-androgenic effects that may impair reproductive and adrenal hormone functions [162]. Pesticides are also suggested to be linked to brain cancer in humans by inducing oxidative stress, damaging DNA, lipids, and proteins. They may disrupt key cellular pathways (e.g., JAK-STAT, Keap1/Nrf2/ARE), impair detoxification, and potentially contribute to the formation of oncometabolites [163].

In aquatic vertebrates, particularly fish, MPs can cause tissue damage such as intestinal inflammation, oxidative stress, and liver toxicity [164]. MPs have been reported to disrupt lipid metabolism and endocrine signaling, impair reproduction, and alter embryonic development [63]. They also may induce gut microbiota imbalance, reducing nutrient absorption and immunity [139]. In seabird chicks, high ingestion of MPs was associated with cell damage and dysfunction of organs such as the stomach, liver, and kidneys, and potentially early neurodegeneration. Despite normal body mass, severe internal harm was observed, indicating that MPs may cause hidden but serious health effects [165]. In humans, MPs enter the body primarily through seafood consumption, inhalation, and skin contact, and may contribute to hormonal imbalances, inflammation, oxidative stress, neurotoxicity, reproductive and developmental disorders, and possibly increased cancer risk [8,164]. In Indo-Pacific humpback dolphins (*Sousa chinensis*), PAHs can accumulate in blubber and might redistribute to vital organs such as the brain during lipid mobilization, potentially increasing the risk of neurotoxicity. Despite low initial levels due to protective barriers, PAHs may contribute to developmental neurotoxicity and reproductive damage [166]. Similarly, consuming PAH-contaminated fish could result in cancer, DNA damage, oxidative stress, hormonal imbalance, immune suppression, and developmental toxicity in humans [167]. A final synthesis of the sources, mechanistic fate, concentration ranges, and the corresponding ecological and human health risks for all six major contaminant groups is comprehensively summarized in Table 3.

## 6. Conclusions

The synthesis presented in this review indicates that bottom sediments are not passive sinks but dynamic biogeochemical systems that can influence the long-term behavior of contaminants. Evidence suggests that interactions among physical, chemical, and microbial processes control whether sediments retain or release pollutants such as nutrients, HMs, PhACs, pesticides, PAHs, and MPs. These processes are strongly affected by redox potential, organic matter composition, and microbial activity. Overall, the findings highlight that pollutant fate in sediments is governed by complex, non-equilibrium mechanisms rather than simple accumulation.

To move from describing these patterns toward predicting them, future research should focus on several specific and testable directions. First, disturbance-driven remobilization thresholds should be quantified by determining how changes in redox potential and sediment resuspension trigger the release of major contaminants like P, As, and PAHs, using controlled microcosms and in situ observations. Second, the competitive sorption kinetics of MPs with organic pollutants need to be characterized under varying pH, salinity, and organic matter conditions, supported by appropriate kinetic and surface complexation models. Third, coupled diagenetic–reactive transport models linking Fe/Mn–S–C cycling with contaminant transformation and stability are needed to improve long-term predictions and guide management under variable environmental conditions. Finally, bioavailability and microbial functionality metrics should be standardized, since total concentration alone does not represent ecological risk; combining in situ passive sampling with metagenomic tools can help link chemical reactivity with biological processes. Addressing these priorities will provide the mechanistic and predictive foundation required for more accurate ecological risk assessment, sustainable sediment remediation, and protection of aquatic ecosystem health under growing anthropogenic and climatic pressures.

## Figures and Tables

**Table 1:** Nutrient concentrations (N & P) and associated pollution sources in bottom sediments from different study locations.

Site and Location	Nitrogen (mg/kg)	Phosphorus (mg/kg)	Pollution Sources	References
Warta River and Tributaries, Poland	71.5–16,240	48.8–8864	urbanization, land use and agricultural activities	[2]
Odra River Estuary (SW Baltic Sea), Poland	200–18,000	80–7940	industrial effluents, fertilizers and untreated agriculture and municipal waste.	[3]
Ogun River, Abeokuta, Nigeria	840–1960	820–2740	wastewater and runoff from residential areas, farmlands, and slaughterhouse (abattoir) discharges	[41]
Marrecas Stream micro basin, Brazil	2806–5233	488–1083	domestic sewage and poultry farm waste	[36]
Lake Chaohu, China	666	509	Sewage, fertilizer runoff	[42]
Three Gorges Reservoir, Yangtze River, China	622–949	916–950	agricultural activities, urban development	[40]

**Table 2:** Microplastics in bottom sediments from selected global sites: polymer types, abundance, size, color, morphology, and likely sources.

Site and Location	Polymer Types	Concentration (mg/kg)	Abundance (Particles/kg)	Size (µm)	Shape/Morphology	Color	Pollution Sources	References
San Pedro Bay, CA, USA	PE, PP, PS, CA, Nylon	416–1102	500–3000	1.73	fibers, foam, fragments, film, tire wear particles	black, gray, blue, clear	textiles, personal care products, urban runoff	[1]
Bay of Bengal, India	PE, PP, PA, PS, PVC, PET	–	80–480	<500	fibers, fragments, films, pellets	blue, yellow, white, black, red, transparent	riverine runoff, fishing gear, packaging	[61]
Bushehr Province (Persian Gulf), Iran	PE, PP, PS, PET, PA	–	660–2140	100–5000	fibers, fragments, pellets, films	black, white, blue, red, gray, transparent	fishing gear, industrial plastics, tourism	[56]
Beibu Gulf (South China Sea), China	CE, PVAL, PE, PP, PS, PET, PU	–	13.12–155.59	330–5000	fibers, fragments, pellets	white, black, transparent, blue, yellow, gray	aquaculture gear, textiles, industrial plastics	[59]
Northern Dvina River, Russia	PE, PP, PET, ABS, PS	52–334	60–650	<100–>300	fibers, fragments, films	gray, transparent, black, red, blue	sewage, industrial discharge, shipyards	[54]
Ontario Lakes, Canada	PE, PP, PET, PA, PVC, PU	–	6–320	42–>5000	fibers, fragments, films, foams, spheres	white, transparent, black, gray, blue	atmospheric deposition, urban runoff, industrial inputs	[14]
Baltic Sea, Northern Europe	PE, PP, PB, PET/PES, PVC	–	103–10,179	200–5000	fibers, films, fragments	transparent, blue, brown, black, white, red, green, yellow	wastewater, maritime transport, urban runoff	[58]

Abbreviations: Polyethylene (PE), polypropylene (PP), polymer blend (PB), polystyrene (PS), polyamide (PA), polycarbonate (PC), polyvinyl chloride (PVC), polyurethane (PUR/PU), polyethylene terephthalate (PET), polyvinyl alcohol (PVAL/PVA), polyester urethane (PU), cellulose acetate (CA), cellulose ester (CE), acrylonitrile butadiene styrene (ABS).

**Table 3:** Risk-Translation Element: Final Synthesis of Sources, Mechanistic Fate, and Toxicological Risk in Bottom Sediments, Synthesized from Literature Cited in this Review (2008–2025).

Pollutant Group	Major Sources	Exposure Pathway (Mechanistic Fate)	Concentration Ranges (mg/kg)	Evidence Strength	Ecological/Human Relevance (Toxicological Endpoints)
Nutrients (N, P)	agricultural runoff, wastewater discharge, animal waste	P is trapped by adsorption to Fe/Al oxides; remobilization (internal loading) occurs when Fe-P bonds disrupt under anoxia and low pH. N is primarily released via microbial breakdown of organic matter (ammonification) and lost to the atmosphere via denitrification.	N (71.5–18,000) and P (48.8–8864)	high	ecological: Triggers eutrophication, leading to hypoxia and mass mortality of benthic life. human: High nitrate concentration in water is associated with health risks, including methemoglobinemia.
HMs (Pb, Cd, Hg, As, Cu, Zn)	industrial and mining effluents, urban runoff, agrochemical inputs	attachment occurs via surface complexation and cation exchange. mobility is entirely controlled by redox speciation: immobilized as metal sulfides (MeS) under anoxia but actively remobilized by competitive desorption and dissolution of carriers (like Fe/Mn oxides) at lower pH.	As (1–46), Cd (0.10–13), Pb (1–272.1), Zn (6–35,300), Hg (0.01–2.40), and Cu (1–298)	high	ecological: Causes Oxidative Stress, enzyme inhibition. human: Bioaccumulation of Methylmercury (MeHg) which is directly linked to severe neurotoxicity.
PhACs	WWTPs effluents, hospital and livestock waste	governed by hydrophobic partitioning (Kow) and ionogenic sorption, which is highly pH dependent. transformation occurs via microbial degradation/conjugation, but their continuous low-level input results in pseudo-persistence.	carbamazepine (0.024–0.3959), diclofenac (0.0089–0.253) and erythromycin (0.038–3.524)	medium	ecological: may cause Endocrine Disrupting Effects (e.g., altered reproduction). human: drives the selection of Antibiotic Resistance Genes (ARGs); evidence suggests mixture effects are a concern.
Pesticides	agricultural runoff, industrial discharge, urban pest control	fate is dominated by strong hydrophobic partitioning to organic matter. while subject to slow microbial mineralization, highly persistent compounds (like some organochlorines) rely on reductive dehalogenation under anoxic conditions, causing them to persist and accumulate in the sediment.	atrazine (0.002–0.183), acetochlor (0.0086–4.315), 2,4-D (0.0391–0.0461)	high	ecological: Acute toxicity, causing acetylcholinesterase inhibition (neurotoxicity). human: May lead to reproductive and hormonal disruption due to endocrine-active ingredients.
PAHs	fossil fuel combustion (pyrogenic), oil spills (petrogenic)	highly hydrophobic; bioavailability is governed by slow desorption from organic carbon. fate involves microbial degradation via dioxygenase enzymes, with high molecular weight PAHs persisting as the primary risk drivers.	0.002–24.3217	high	ecological: Acute toxicity, causing acetylcholinesterase inhibition (neurotoxicity). human: May lead to reproductive and hormonal disruption due to endocrine-active ingredients.
MPs	plastic-waste degradation, wastewater discharge, maritime and fisheries activities	physical fate: transport is governed by particle density and shape. sedimentation is accelerated by biofouling (attachment of microbes/organic matter). chemical Fate: acts as a vector (via surface adsorption) for hydrophobic co-contaminants (like PAHs and HMs), facilitating their trophic transfer upon ingestion.	52–1102	emerging	ecological: High potential for mutagenicity and carcinogenicity (via AhR pathway). human: Consumption of contaminated food is associated with increased cancer risk.

Abbreviations: nitrogen (N), phosphorus (P), microplastics (MPs), polycyclic aromatic hydrocarbons (PAHs), pharmaceutical active compounds (PhACs), heavy metals (HMs) lead (Pb), cadmium (Cd), mercury (Hg), arsenic (As), copper (Cu), zinc (Zn), wastewater treatment plants (WWTPs), octanol-water partition coefficient (Kow).

## Data Availability

All relevant information and literature sources are included within this manuscript. Additional data or supporting materials can be provided by the corresponding author upon reasonable request.
